# P-1746. Use of Intrathecal Amphotericin for Coccidioidal Meningitis

**DOI:** 10.1093/ofid/ofaf695.1917

**Published:** 2026-01-11

**Authors:** Rebecca Y Linfield, Eva Gorenburg, Jane W Liang, Guillermo Rodriguez-Nava, Vanessa El Kamari, Julie Parsonnet

**Affiliations:** Stanford University, Stanford, California; Stanford University, Stanford, California; Kaiser Permanente Northern California, Pleasanton, California; Stanford University School of Medicine, Stanford, California; Stanford University, Stanford, California; Stanford University, Stanford, California

## Abstract

**Background:**

Cases of coccidioidomycosis are increasing dramatically in the U.S. The utility of intrathecal (IT) amphotericin in treating coccidioidal meningitis (CM) in the era of azole therapy is unknown. We sought to understand how IT therapy is associated with mortality.

**1A:**
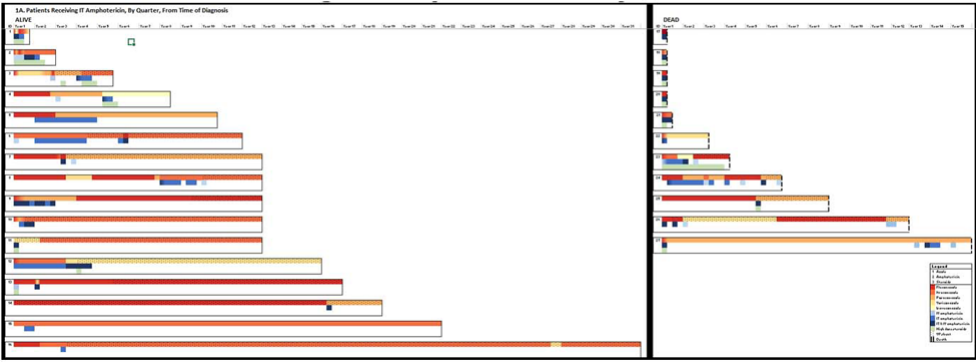
Patients Receiving IT Amphotericin, by Quarter, From Time of Diagnosis

**1B:**
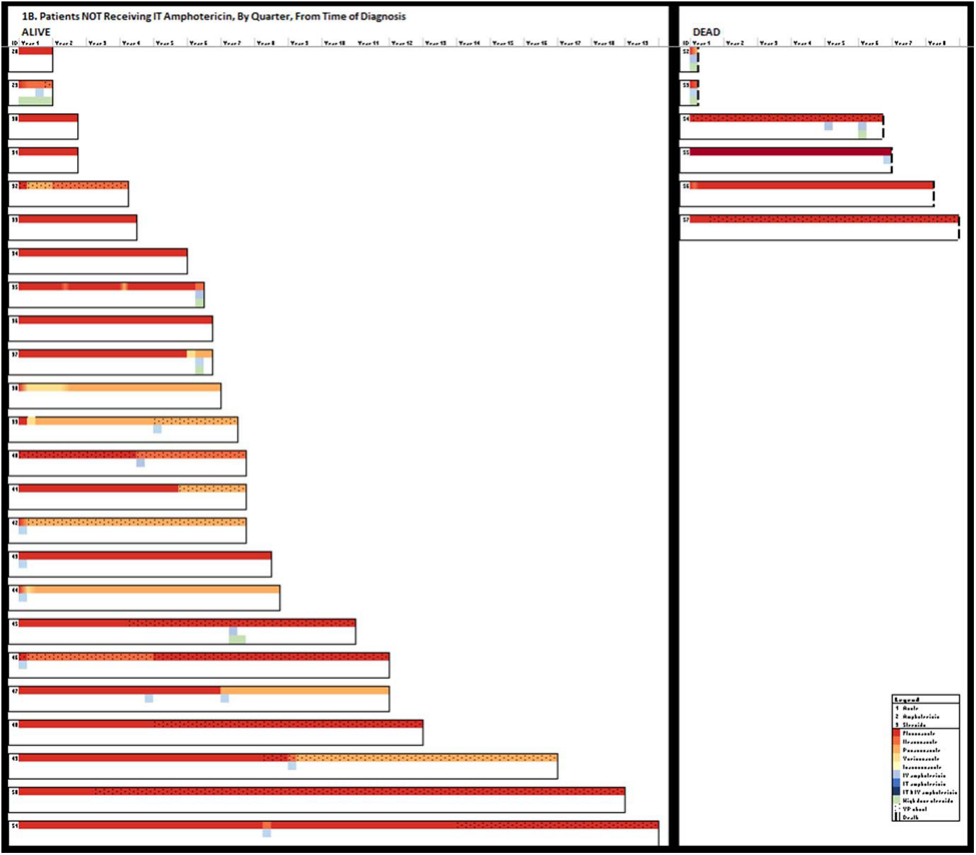
Patients NOT Receiving IT Amphotericin, By Quarter, from Time of Diagnosis

**Methods:**

We conducted a retrospective chart review of adult patients with laboratory-proven CM seen at Stanford Hospital and Clinics by an Infectious Diseases physician from 2008-2023.

**Table 1:**
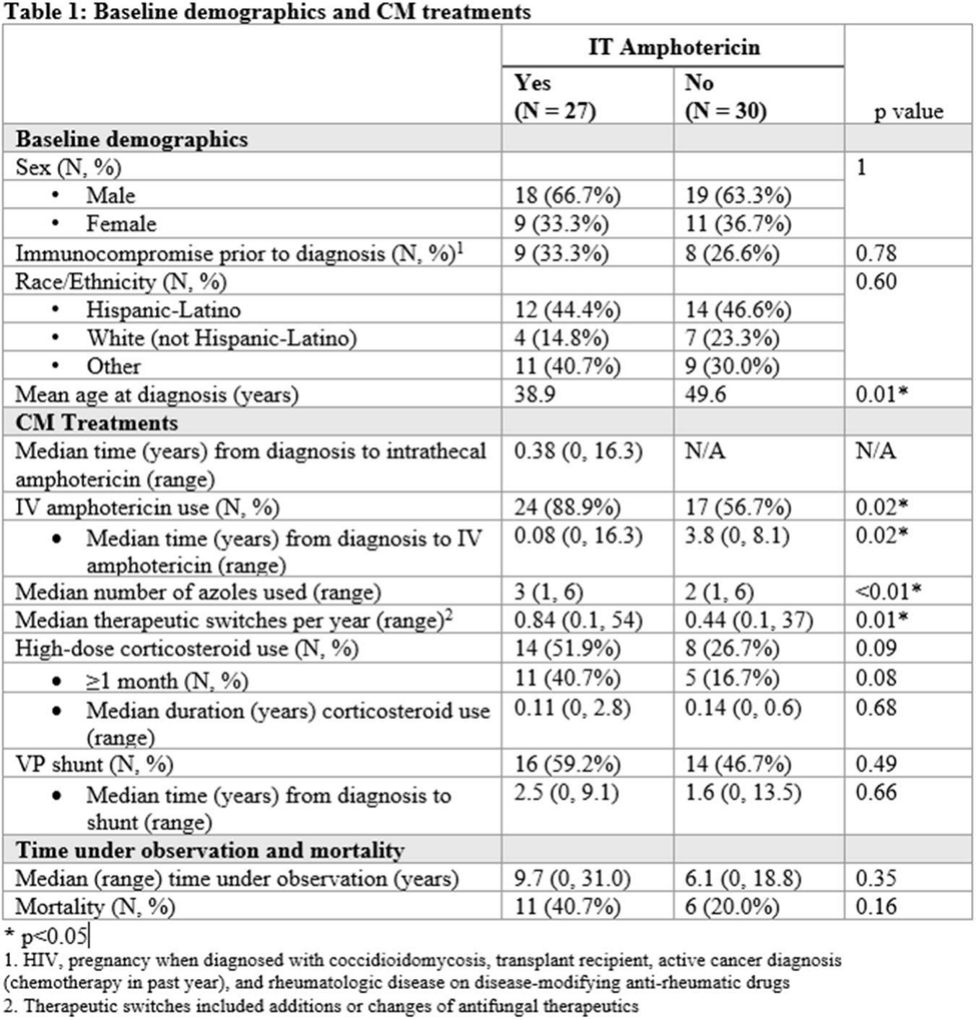
Baseline demographics and CM treatments

**Table 2:**
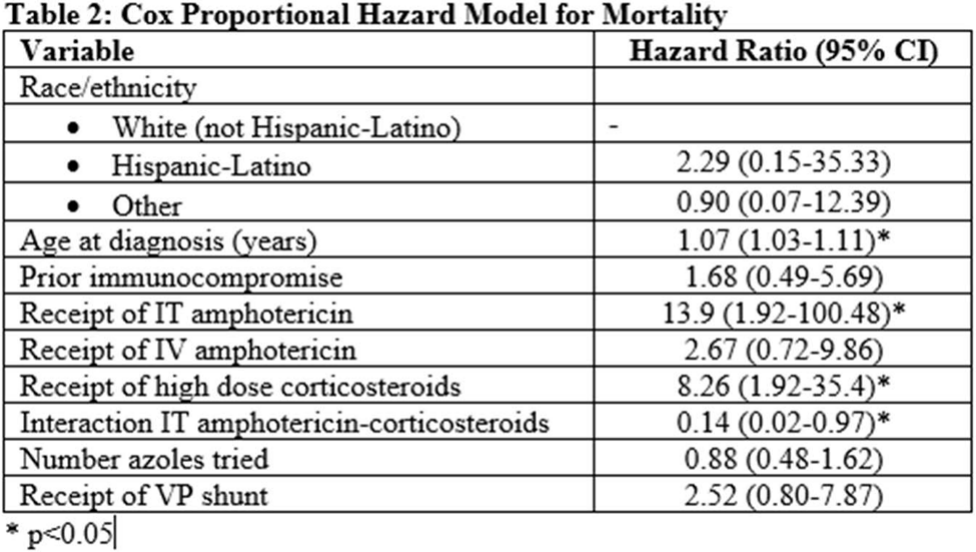
Cox Proportional Hazard Model for Mortality

**Results:**

57 patients met inclusion criteria. All patients received azole therapy. Twenty-seven of those patients (47.4%) additionally received IT amphotericin. Patients receiving IT therapy had higher rates of IV amphotericin use (88.9% vs. 56.7%, p = 0.02) and corticosteroid use (51.9% vs. 26.7%, p = 0.09), and higher median therapeutic switches per year (0.84 vs. 0.44, p = 0.01). An unadjusted Kaplan-Meier curve demonstrated a five-year mortality rate of 26.7% in the IT amphotericin group, compared to 6.7% in non-receiver group, p = 0.28. A Cox regression revealed that older age at diagnosis (HR 1.07, 95% CI 1.03-1.11), receipt of IT amphotericin (HR 13.9, 95% CI 1.93-100.48), and corticosteroid use (HR 8.3, 95% CI 1.92-35.4) were significantly associated with mortality, with a significant interaction between IT amphotericin and corticosteroids (interaction HR 0.14, 95% CI 0.02-0.97).

**Conclusion:**

Patients who received IT amphotericin had higher mortality rates than those who did not, likely reflecting disease refractory to treatment. More therapies are needed for those who have disease progression on azole therapy.

**Disclosures:**

All Authors: No reported disclosures

